# SIV antigen immunization induces transient antigen-specific T cell responses and selectively activates viral replication in draining lymph nodes in retroviral suppressed rhesus macaques

**DOI:** 10.1186/1742-4690-8-57

**Published:** 2011-07-13

**Authors:** Haitao Hu, Lucio Gama, Pyone P Aye, Janice E Clements, Peter A Barry, Andrew A Lackner, Drew Weissman

**Affiliations:** 1 Division of Infectious Diseases, University of Pennsylvania, Philadelphia, PA, USA; 2 Department of Molecular and Comparative Pathobiology, Johns Hopkins University School of Medicine, Baltimore, MD, USA; 3 Tulane National Primate Research Center, Covington, LA, USA; 4 Center for Comparative Medicine, University of California-Davis, Davis, CA, USA

## Abstract

**Background:**

HIV infection causes a qualitative and quantitative loss of CD4^+ ^T cell immunity. The institution of anti-retroviral therapy (ART) restores CD4^+ ^T cell responses to many pathogens, but HIV-specific responses remain deficient. Similarly, therapeutic immunization with HIV antigens of chronically infected, ART treated subjects results in poor induction of HIV-specific CD4 responses. In this study, we used a macaque model of ART treatment during chronic infection to study the virologic consequences of SIV antigen stimulation in lymph nodes early after immunization. Rhesus CMV (RhCMV) seropositive, Mamu A*01 positive rhesus macaques were chronically infected with SIVmac251 and treated with ART. The immune and viral responses to SIV gag and RhCMV pp65 antigen immunization in draining lymph nodes and peripheral blood were analyzed. Animals were immunized on contralateral sides with SIV gag and RhCMV pp65 encoding plasmids, which allowed lymph nodes draining each antigen to be obtained at the same time from the same animal for direct comparison.

**Results:**

We observed that both SIV and RhCMV immunizations stimulated transient antigen-specific T cell responses in draining lymph nodes. The RhCMV-specific responses were potent and sustained (50 days post-immunization) in the periphery, while the SIV-specific responses were transient and extinguished quickly. The SIV antigen stimulation selectively induced transient SIV replication in draining lymph nodes.

**Conclusions:**

The data are consistent with a model whereby viral replication in response to SIV antigen stimulation limits the generation of SIV antigen-specific responses and suggests a potential mechanism for the early loss and poor HIV-specific CD4^+ ^T cell response observed in HIV-infected individuals.

## Background

CD4^+ ^T cells play a central role in maintaining effective cellular and humoral immune responses by providing help to CD8^+ ^T cells, B cells and innate effectors. The protective role of CD4^+ ^T cell responses in HIV-1 infection has been suggested in previous studies [1,2]. However, HIV-1 infection results in the progressive loss of CD4^+ ^T cell responses, which is characterized as both a decline in the number of CD4^+ ^T cells and a loss of the functional activity of cells with certain antigenic specificities [3-6]. Although the institution of anti-retroviral therapy (ART) causes viral suppression and recovery of CD4^+ ^T cell response to some common pathogens, HIV-specific CD4 response remains deficient [7,8]. Similarly, immunization of chronically infected, ART treated patients with HIV antigens does not result in the generation of significant HIV-specific CD4^+ ^T cell responses, suggesting that HIV-specific CD4+ T cells are dysfunctional or preferentially depleted in infection and fail to recover [9-11]. The mechanisms for the failure of HIV antigen immunization to induce HIV-specific CD4^+ ^response are not fully clear [12].

During an immune response, antigen-presenting cells (APC) activate CD4^+ ^T cells to specific antigen specificities in lymphoid tissue. However, in HIV-1 infection, lymphoid tissue also represents an important site for viral replication, and the interaction between APC and CD4^+ ^T cells may enhance viral replication by multiple mechanisms (reviewed in [13]). It has been shown that even in the setting of potent regimens of ART, a very low level of viral replication could still be detected [14-16], which may be derived from DC mediated activation of latent virus in memory CD4^+ ^T cells, homeostatic regulation of memory populations, or other long-lived reservoirs [17]. Given that HIV-specific CD4^+ ^memory T cells are preferentially infected by HIV, carrying more viral DNA than total memory cells [18], we were interested in determining if activation of HIV-specific CD4^+ ^T cells results in viral replication in lymphoid tissue.

We used a rhesus macaque model of ART treatment during chronic infection to study the effects of SIV antigen stimulation in lymph nodes (LNs) compared to a control immunogen on viral replication early after immunization. Mamu A*01^+ ^rhesus macaques were infected with SIVmac251 and after 4 months treated with ART resulting in viral suppression and immune reconstitution. The macaques were also rhesus CMV (RhCMV) seropositive and immunized with an RhCMV immunogen as a control antigen stimulation. Animals were immunized on the left side (both arms and legs) with an SIV gag-encoding expression plasmid and on the right side (both arms and legs) with a RhCMV pp65-encoding expression plasmid, which allowed draining LNs for each antigen to be obtained from the same animal at the same time, allowing for a direct comparison of the effect of SIV and RhCMV antigen stimulation on viral replication.

## Results

### Infection and immunization of rhesus macaques

**Figure 1:**
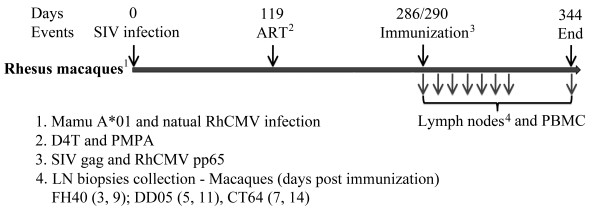
**Experimental protocol for infection and immunization of rhesus macaques**. Mamu A*01 rhesus macaques naturally infected with RhCMV were intravenously inoculated with SIVmac251 followed by ART treatment (DT4 and PMPA) from days 119 post infection through the end of experiment. On days 286 or 290 post infection, monkeys received immunizations with SIV gag encoding DNA i.m. (2 mg per injection), in the left arm and left leg, and immunizations with RhCMV pp65 encoding DNA i.m. (2 mg per injection) in the right arm and leg. LNs biopsies draining either SIV gag or RhCMV pp65 immunization sites from the same animal at the same time were obtained on Day 3 for FH40, Day 5 for DD05, Day 7 for CT64, Day 9 for FH40, Day 11 for DD05, and Day 14 for CT64. LN biopsies from both sides were also collected from all animals on Day 60 post immunization. PBMCs were collected pre-immunization and every 2-3 weeks post immunization.

All animals used in this study (FH40, DD05, and CT64) were Mamu A*01 positive to reduce MHC variation in disease course and T cell responses and were naturally infected with RhCMV. The study was designed to infect animals (1000 TCID_50 _of SIVmac251 by intravenous injection) and allow them to reach steady state viral loads (4 months) followed by ART treatment (PMPA and D4T) for 5.5 months. Animals were then immunized with plasmids encoding SIV gag or RhCMV pp65 in both arms and legs. Two LN biopsies draining either SIV gag or RhCMV pp65 injections from the same animal at the same time were obtained on the indicated days (D3: FH40 Inguinal; D5: DD05 Inguinal, D7: CT64 Inguinal; D9: FH40 Axillary; D11: DD05 Axillary, D14: CT64 Axillary) (Figure 1). Immunizations used expression plasmids previously used as vaccines that were demonstrated to induce potent T cell responses in uninfected rhesus macaques [19-25]. Serum and PBMCs were obtained every 2 to 3 weeks throughout the experiment.

### ART suppresses viral replication with recovery of peripheral CD4+ T cell counts

**Figure 2:**
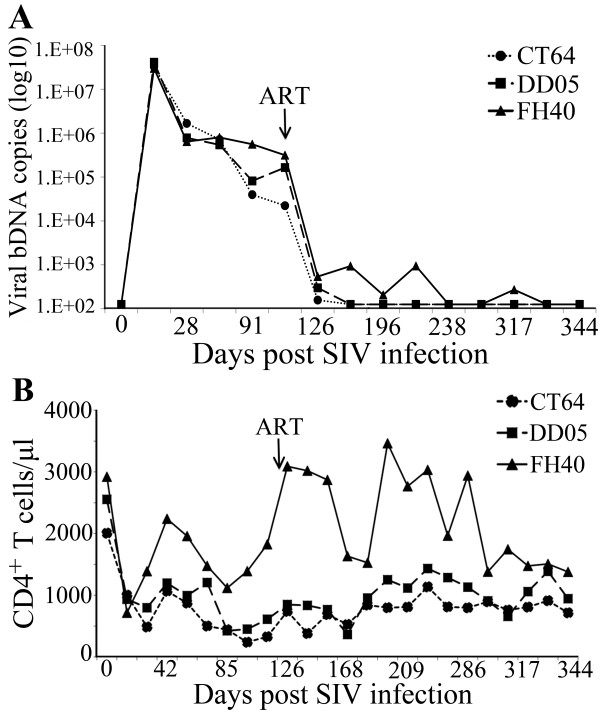
**Viral loads and peripheral CD4^+ ^T cell counts**. Viral loads and CD4 counts were measured every 2 to 3 weeks throughout the experiment. (A) Viral RNA in plasma was quantified by a bDNA signal amplification assay and expressed as viral RNA copies per ml plasma. ART treatment controlled the viral loads in all three animals. (B) Peripheral CD4^+ ^T cell counts increased after the initiation of ART. Macaque blood samples were stained for CD3, CD4, and CD8 and the number of CD3^+^, CD4^+ ^T lymphocytes were determined by flow cytometry and peripheral white blood cell counts. CD4^+ ^T cell counts are expressed as CD4^+ ^T cells per μl blood.

SIV infection was established in all three animals with kinetics typical of primary infection in naïve rhesus macaques (Figure 2A) [26,27]. Introduction of ART 4 months post infection, when set point viral loads had been established, efficiently suppressed viral replication to undetectable levels. One macaque demonstrated occasional blips in viral load that returned to undetectable levels by the subsequent measurement without any change in therapy (Figure 2A). Absolute CD4^+ ^T cell counts in the peripheral blood demonstrated stabilization after introduction of ART in all animals with sustained levels of more than 500 cells/μl (Figure 2B). These findings demonstrate that chronic SIV infection was achieved and ART successfully suppressed viral replication leading to partial recovery of peripheral CD4^+ ^T cell counts.

### SIV and RhCMV antigen immunization induces antigen-specific T cell responses in draining LNs

**Figure 3:**
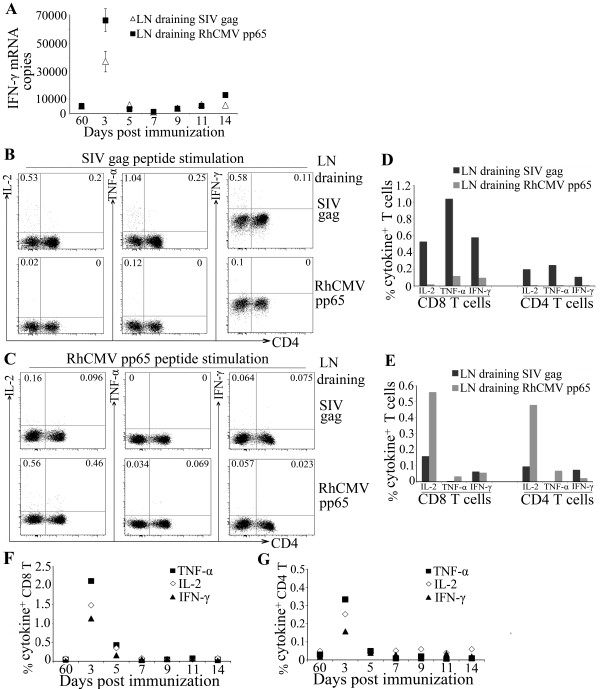
**Antigen-specific responses in LNs draining SIV gag and RhCMV pp65 immunization sites**. (A) Composite result for IFN-γ mRNA levels in LNs draining SIV gag or RhCMV pp65 immunization sites for each animal on indicated time points. Total RNA from LNMCs was collected on the indicated days post immunization and subject to real-time PCR for the quantitation of IFN-γ mRNA. The results are expressed as the number of copies of IFN-γ mRNA per μg total RNA. Standard deviation of the mean for triplicate analyses for Day 3 LNs draining SIV gag and RhCMV immunization sites are shown. (B and D) Intracellular cytokine staining (ICS) for SIV gag-specific CD4^+ ^and CD8^+ ^T cell responses in day 3 LNMCs. Day 3 post immunization LNMCs draining either SIV-gag (top panels, B) or RhCMV-pp65 (bottom panels, B) immunization sites were stimulated with SIV gag 15-mer overlapping peptide pools and stained for IL-2, TNF-α, and IFN-γ. SIV-specific cytokine-producing CD4^+ ^and CD8^+ ^T cells were analyzed by multi-color flow cytometry. (C and E) Intracellular staining of RhCMV pp65-specific CD4^+ ^and CD8^+ ^T cell responses in day 5 LNMCs. Day 5 post immunization LNMCs draining either SIV-gag (top panels, C) or RhCMV-pp65 immunizations (bottom panels, C) were stimulated with RhCMV pp65 15-mer overlapping peptide pools and stained for IL-2, TNF-α, and IFN-γ. The composite results for percent of SIV-specific cytokine producing CD8^+ ^(F) and CD4^+ ^(G) T cells in LNMCs draining SIV gag immunization sites for one macaque at indicated days post immunization are shown. Cytokine producing T cells in LNMCs without stimulation (background) were subtracted from all flow analyses.

Animals were immunized with SIV gag encoding plasmid in the arm and leg on one side and RhCMV pp65 encoding plasmid on the other side at the same time. LNs, one draining an SIV immunization site and one draining an RhCMV immunization site, were excised from each animal at 2 time points (axillary and inguinal) post immunization. LN biopsies from both sides from all macaques were also collected on day 60-post immunization. We found that the levels of antigen-specific responses in day 60 LNs were similar in comparing both antigens in both LNs, suggesting that the effects of immunization on T cell responses in the LNs were transient and returned to baseline by 60 days post immunization (Figure 3 and data not shown). Therefore, this time point was chosen as a baseline for standardization.

First, we assessed immune activation induced by antigen immunizations in draining LNs to determine whether the two DNA plasmids were immunogenic. LN mononuclear cells were analyzed for IFN-γ mRNA expression, a major effector cytokine for adaptive immunity. As shown in Figure 3A, an increase in IFN-γ mRNA expression was detected in LNs draining SIV gag and RhCMV pp65 on day 3 post immunization, which was followed by a decline on day 5 to the baseline levels as observed in day 60 LNs (Figure 3A). The result suggests that both DNA plasmids are immunogenic in the rhesus macaques used in this study, which is consistent with previous primate studies where the same DNA plasmids were used and shown to be immunogenic in uninfected macaques [19-25].

We then evaluated the antigen-specific T cell responses in LNs by measuring ex vivo cytokine production of LN mononuclear cells. Cells were stimulated with either SIV gag or RhCMV pp65 peptide pools and production of IL-2, TNF-α and IFN-γ in T cells was determined by polychromatic flow cytometry. Flow cytometry plots for cytokine staining are shown (Figure 3B and 3C). We found that the LN draining SIV gag on day 3, when the immune activation, as measured by IFN-γ mRNA, was the highest, demonstrated potent gag-specific T cell responses based on IL-2, TNF-α and IFN-γ production (Figure 3B and 3D). In contrast, the day 3 LN draining RhCMV pp65 immunization from the same animal, when stimulated by gag peptides, demonstrated no significant response (Figure 3B and 3D). Some multifunctionality of the CD4^+ ^T cell response was observed with approximately 4% expressing three cytokines and 28% expressing two. Similarly, the LNs collected on day 5 post immunization were evaluated for RhCMV-specific T cell responses by stimulating the LN cells with RhCMV-pp65 peptides. A significant RhCMV-specific response was induced in the LN draining the RhCMV pp65 compared to the LN draining the SIV gag immunization site (Figure 3C and 3E). Draining LN responses to SIV immunization decreased by day 5 and from day 7 onwards were similar to the levels observed in day 60-post immunization LNs (Figure 3F and 3G). Taken together, these data suggest that both SIV and RhCMV immunization induced transient antigen-specific T cell responses in draining LNs.

### Differential SIV- and RhCMV-specific T cell responses in peripheral blood

**Figure 4:**
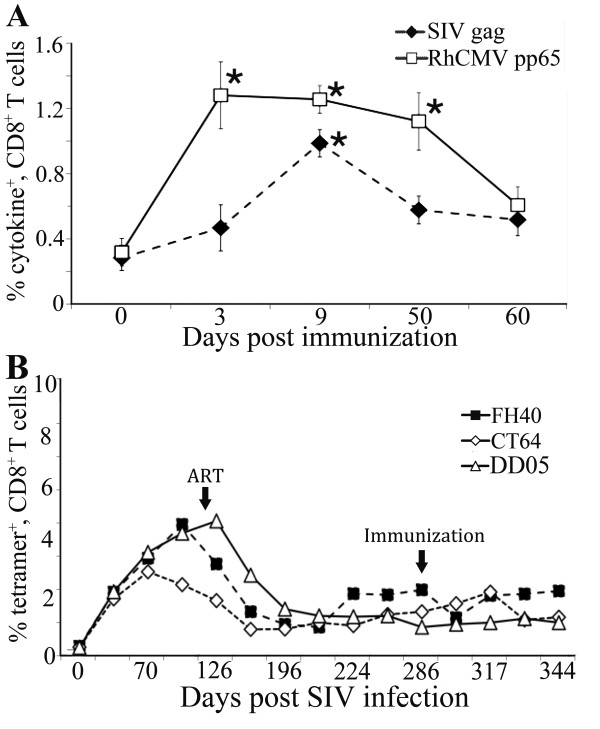
**SIV- and RhCMV-specific T cell responses in peripheral blood**. (A) PBMCs before and after immunizations were stimulated with either SIV gag or RhCMV pp65 peptide pools and stained for IL-2, TNF-α, and IFN-γ. The percentages of cytokine-producing CD8^+ ^T cells were determined by multi-color flow cytometry. The percent of CD8^+ ^T cells able to make any cytokine is shown. The average of all animals is shown on the indicated days post immunization. Error bars are the standard error of the mean, p-values using the student's t-test comparing the levels before immunization (Day 0) to time points post-immunization are shown as a * indicating a *p *< 0.05. (B) Measurement of p11c tetramer^+^, CD8^+ ^T cells throughout the experiment. The results are expressed as percent of tetramer^+^, CD8^+ ^T cells on indicated days post SIV infection for each animal.

Both antigens were immunogenic and induced antigen-specific T cell responses in draining LNs, we next analyzed T cell responses induced by immunization in the peripheral blood. PBMC collected at multiple time points post immunization were stimulated with SIV gag or RhCMV pp65 peptide pools and the frequency of cytokine producing T cells were analyzed by polychromatic flow cytometery. We expressed the data as the percent of CD8^+ ^T cells able to produce any cytokine or combination of cytokines (IL-2, TNF-α and/or IFN-γ) after subtracting the levels in unstimulated cells. The average of all animals is shown (Figure 4A). A potent increase in RhCMV-specific CD8^+ ^T cells was observed at day 3-post immunization in blood with an average of 1.4% of cells able to produce a cytokine. The RhCMV-specific response was sustained until day 50 post-immunization. In contrast, SIV-specific CD8^+ ^T cell responses were transient and extinguished quickly in the blood with only an increase on day 9 post-immunization (Figure 4A). Further characterization of the gag and pp65 responses demonstrated that gag-specific cells had increased levels of expression of PD1 and contained both central memory (CD95^+^, CD28^+^) and effector memory (CD95^+^, CD28^-^) cells, while the pp65 responding cells were predominantly effector memory phenotype. All animals demonstrated similar kinetics of gag and pp65 specific responses. PBMCs were stained with the Mamu A*01 specific gag tetramer, p11C (CTPYDINQM). All animals demonstrated a decrease in tetramer positive CD8^+ ^T cells after the initiation of ART, but levels remained above 1% after 5.5 months of suppressive ART (Figure 4B).

### SIV antigen immunization induces transient activation of viral replication in the draining LN

**Figure 5:**
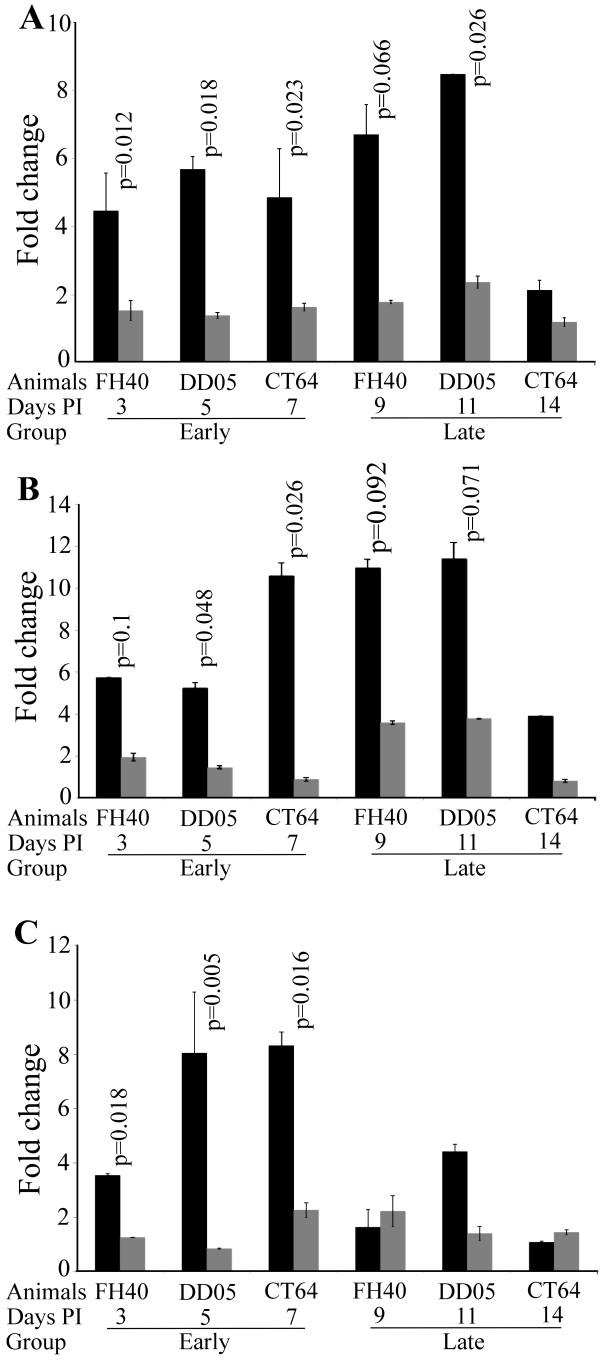
**Quantitation of viral RNAs in LNMCs draining immunization sites**. Total RNA extracted from LNMCs draining either SIV gag or RhCMV pp65 immunization sites, one animal per time point, were subject to real-time PCR to quantitate SIV doubly spliced (tat) (A), singly spliced (vif) (B), and unspliced (gag) (C) RNA. LNMCs draining the RhCMV pp65 immunization site (Gray). LNMCs draining the SIV gag immunization site (Black). Copy numbers of SIV RNAs were normalized to macaque GAPDH mRNA, and the results for each time point following immunization (PI, post immunization) are shown as fold change relative to RNA analyzed on day 60-post immunization from the same animal, as no differences between LNs draining gag or pp65 immunizations were found at this time point. Error bars are the standard error of the mean of replicate analyses. Statistical analysis compared viral RNA between LNMCs draining SIV and CMV immunization sites in the same animal on the same day. Most measurements were repeated in at least two separate experiments with identical results.

We next determined whether immunization with SIV or CMV antigens induced the activation of viral replication at early, 3 to 7 days, or late, 9 to 14 days, time points. Total RNAs from LN mononuclear cells (LNMC) draining either SIV gag or RhCMV pp65 immunization sites were analyzed by real-time PCR for early and late SIV RNA transcripts, including doubly spliced (tat), singly-spliced (vif), and unspliced (gag) RNA [28]. The use of isolated cells with multiple washes both before and after cryopreservation removed any extracellular viral RNA that was present as free or germinal center associated virions. All comparisons were made between LNs from the same animal obtained at the same time that differed only in whether they drained an SIV or a RhCMV immunization site. We first investigated SIV RNAs in day 60 LNMCs and found low levels of all 3 transcripts. Importantly, no difference was observed for each viral RNA species at this time point in comparing LNMCs draining SIV and RhCMV immunization sites from the same animal. Therefore, we used these levels of SIV RNA as a baseline for the analysis of the effect of antigen stimulation on viral replication for that animal. Copy numbers of SIV RNA were normalized to GAPDH RNA, and the results for each time point following immunization are shown as fold change relative to RNA collected at 60 days post immunization for that animal (Figure 5).

Each analysis was performed in the same animal at the same time point where the only difference was the type of immunization drained, thereby avoiding the need to consider confounders present in comparisons between animals. The effects of antigen-specific stimulation on SIV replication were first determined by comparing the levels of SIV doubly spliced RNA in LNMCs draining SIV gag and RhCMV pp65 immunization. Doubly spliced RNA copies were significantly increased in the LNMCs draining the SIV gag immunization compared to those draining the pp65 immunization on days 3, 5, and 7 (Figure 5A). Singly spliced SIV RNA was increased in LNMCs draining SIV gag immunizations on days 5 and 7 (Figure 5B). Similar to the spliced SIV RNAs, all three monkeys demonstrated a statistically significant increase in unspliced viral RNA in LNMCs draining SIV gag immunization compared to RhCMV-pp65 immunization on days 3, 5, and 7 (Figure 5C). At the latter time points, viral replication in the LNMCs draining SIV gag immunizations were not significantly increased compared to LNMCs draining RhCMV pp65 and by day 14, all viral RNAs from LNMCs draining both gag and RhCMV decreased towards baseline levels (Figure 5 A-C). Random effects models were used to adjust for observations from the same animal, which demonstrated that on days 3-7, there was a statistically significant increase in viral replication comparing LNMCs draining SIV gag to those draining RhCMV pp65.

We hypothesized that viral replication would occur soon after immunization and analyzed the early (days 3, 5, and 7) and late (days 9, 11, and 14) time points as groups. Comparing viral replication in all animals induced by gag and pp65 immunization in draining LNMCs at the early time point (days 3 - 7) showed a significant increase by gag immunization; doubly spliced (p = 0.0007), singly spliced (p = 0.029), and unspliced (p = 0.032). These data show that all rhesus macaques demonstrated significant increases, whether analyzed singly or as a group, in viral replication in LNMCs draining SIV gag immunizations. The percent of CD4+ T cells in LNMCs did not differ between LNs draining SIV gag and RhCMV pp65 immunization sites, suggesting that the increase in viral gene expression was not due to differential CD4^+ ^T cell migration to LNs draining gag immunization sites, although we cannot rule out that a larger fraction of cells that trafficked to the gag LN were infected.

### No increase in viral load was observed in peripheral blood in response to immunization

We next sought to determine whether this transient viral replication in LNMCs was observed in peripheral blood. Viral loads, measured by standard bDNA signal amplification, in peripheral blood prior to and after antigen immunization in all animals are shown in Table 1. Except for a transient viral blip in one animal at day 31 post-immunization (270 copies/μl) that returned to undetectable 3 weeks later, no increase in viral loads that could be attributed to SIV gag immunization induced viral replication was detected. The data suggest that in the setting of potent anti-viral suppression, SIV antigen immunization activated viral replication was transient and restricted to draining LNs without spread to the periphery.

**Table 1:** Viral loads in periphery during antigen immunization

Days PI*	238	290	317	336	344
Animal					
CT64	< 125	< 125(D0*)	< 125(D27*)	< 125	< 125
DD05	< 125	< 125(D0*)	< 125(D27*)	< 125	< 125
FH40	< 125	< 125(D4*)	270(D31*)	< 125	< 125

* Days post immunization.

## Discussion

A biphasic destruction of CD4^+ ^T cells is observed in HIV infection with a massive loss of CD4^+ ^T cells during early infection and a subsequent progressive loss during the chronic stage of infection [29,30]. Retroviral suppression by ART results in an increase in peripheral CD4^+ ^T cell counts and functional reconstitution of CD4^+ ^T cell responses to many common antigens [31,32], but HIV-specific CD4^+ ^T cell responses remain deficient [33]. We studied ART-treated, chronic SIV and RhCMV infected rhesus macaques and observed; 1) that both SIV and RhCMV antigen immunizations could induce immune activation and antigen-specific T cell responses in draining LNs. 2) In peripheral blood, the RhCMV-specific response induced by immunization was potent and sustained, whereas the SIV-specific response extinguished quickly. 3) We observed that SIV antigen immunization transiently induced greater levels of SIV replication in draining LNs of all animals compared to RhCMV immunization. In this study, the experimental design of immunizing the same animal with both an SIV antigen and a non-SIV antigen on collateral sides allowed us to directly compare the early immune and viral responses in draining LNs from the same animal at the same time, making it possible to study the effect of antigen stimulation in the context of ART-treated, chronic infection with limited animal numbers. All 3 animals displayed persistent CMV specific T cell responses for 50 days and demonstrated a weak transient SIV specific T cell response in peripheral blood. LNMCs from all animals demonstrated elevated levels of viral replication in response to SIV antigen immunization during the first 7 days after immunization. The data do not prove a causal link between the weak SIV T cell responses and LN viral replication, but are consistent with a hypothesis that SIV antigens induce viral replication that leads to depletion or dysfunction of antigen specific cells leading to a reduction in the strength and longevity of the response.

Pathogenic SIVmac251 infection of rhesus macaques has been well described as one of the preferred experimental models for studying HIV pathogenesis [34]. In this study, all rhesus macaques inoculated with SIVmac251 were Mamu A*01 positive to control for an MHC effect on viral immune responses and disease progression, as well as to aid in the measurement of immune responses by a Mamu A*01 restricted SIV gag tetramer. All macaques established primary SIV infection with typical viral replication dynamics [26,27,35], and demonstrated responsiveness to ART with rapid control of viremia (Figure 2A). One animal had occasional blips in viral load that returned to undetectable without changes in therapy. It has been shown that even during the most potent regimens of retroviral suppression, the presence of virus in plasma could be measured by some ultrasensitive assays [14-16]. We believe the blips in FH40 are similar to those observed during therapy in humans, which are not associated with acute infection of cells [36] and believe that this is representative of a range of low level virus that can be measured during ART [37]. One study identified that HIV-infected subjects that developed blips in viral load had higher instead of lower levels of CD4^+ ^T cell responses to gag [38]. We do not believe that the macaque with blips in viral load is responding differently to ART compared to the animals without measureable blips.

Therapeutic immunization for HIV infection during ART has been studied in SIV-infected rhesus macaques and the immunological and virologic consequences have been investigated in peripheral blood using DNA immunization [39-42], as well as other systems [43-45]. After release from ART, variable immunologic and virologic benefits were reported from no control [42], temporal control [43], to long-lasting virologic control [44]. In this study, using RhCMV immunization as a non-SIV control in the same animal, we investigated the immunologic and virologic consequences of immunization with SIV antigen in chronic SIV and RhCMV co-infected, ART treated rhesus macaques focusing on the early response in draining LNs. The plasmids encoding immunogens used for immunization were previously demonstrated to induce potent immune responses in uninfected macaques [19-25]. The RhCMV pp65 plasmid had a greater number of immunostimulatory motifs, which would bias towards the null hypothesis. Moreover, all the comparisons between LNs at each time point were from the same animal collected at the same time, thus allowing us to use each animal as its own control. Also, our study focused on the local responses in draining LN, where the antigen-specific T cell responses and viral replication occur, rather than systemic responses in peripheral blood, as done in most previous studies. In addition, we chose a range of days post immunization covering the early activation of memory T cells and generation of effector responses. Our data show that both SIV and RhCMV antigen immunizations induced transient immune activation and antigen-specific T cell responses in draining LNs. Further measurement of these responses in peripheral blood showed that the RhCMV-specific responses were sustained in PBMC with a rapid onset of cytokine producing CD8^+ ^T cells 3 days post immunization, which was maintained at day 50-post immunization, whereas the immunization induced SIV-specific T cell responses were transient, appearing only on day 9 post-immunization, and extinguished quickly in blood. The peripheral SIV- and RhCMV-specific CD8^+ ^T cell responses were significantly induced compared to pre-immunization levels, supporting that both immunizations induced immune responses. Of interest, a study that repeatedly immunized macaques with long-standing ART-treated SIVmac251 infection induced stronger SIV-specific CD4+ and CD8^+ ^T cell responses in blood [46]. This study used the MVA vector and delivered three immunizations, whereas in our study, only a single gag DNA immunization was used.

HIV-specific CD4+ memory T cells are preferentially infected by HIV, carrying approximately 2- to 5-fold more viral DNA than total memory cells [18]. We hypothesize that during an HIV-specific response, activation of HIV-specific CD4+ T cells, which bear higher amounts of latent virus, results in activation of viral replication in the LN leading to suppression of CD4^+ ^T cells and the HIV-specific responses through multiple mechanisms, while a non-HIV antigen-specific response activates less viral replication allowing more efficient expansion of the response.

## Conclusions

We used a naturally RhCMV and experimentally SIVmac251 co-infected and ART treated rhesus macaque model to study the effects of immune stimulation with SIV gag and RhCMV pp65 antigens. The study concentrated on the early immune and viral responses in draining lymphoid organs. Both antigen immunizations were able to induce transient immune activation and antigen-specific T cell responses in draining LNs, but the SIV gag immunization also induced significant viral replication. Following immunization, the SIV response extinguished quickly in peripheral blood, while the RhCMV response was sustained. Our data suggest that SIV antigens, as part of the normal immune response to the virus, leads to T cell stimulation that could potentially lead to viral replication resulting in an impairment in the generation of virus specific CD4^+ ^T cells. It is possible that this mechanism is responsible for the observation that in progressing HIV-infected subjects, CD4-specific responses to HIV antigens are lost early in infection and are difficult to restore or induce. Further studies are needed to determine if there is a causal link between SIV or HIV antigen induced viral replication and impairment of CD4^+ ^T cell responses to the virus.

## Methods

### Ethics statement

All animal experiments were performed in strict accordance with the standards of the Association for Assessment and Accreditation of Laboratory Animal Care International and the "Guide for the Care and Use of Laboratory Animals" prepared by the National Research Council. The studies were approved by the University of Pennsylvania and Tulane Institutional Animal Care and Use Committees.

### Immunogens

Plasmid DNA expressing the SIV Gag core protein from SIVmac239 (pSIVgag) was used. It is a Rev-independent expression vector designed for a high level of protein expression, as previously described [19,21-23]. Protein expression is under the transcriptional control of the immediate-early promoter/enhancer of human CMV and the bovine growth hormone polyadenylation signal. Plasmid DNA expressing the RhCMV pp65 protein (pND/pp65-2) was used [20,24,25]. The expression of RhCMV pp65 uses the same promoter and polyadenylation signal. The GC and CpG content of the plasmids with inserts are: pSIVgag, 40.5% GC, no CpG-S (GACGTT or AACGTT) motifs and 0.03 potential CpG-N motifs (CCG, CCGG, CGG) per bp; pND-pp65, 51.3% GC, 3 CpG-S motifs and 0.0344 potential CpG-N motifs per bp. Neither contained any human optimal TLR9 immunostimulatory motifs (TGTCGTT). DNA was formulated for injection in 0.15 M citrate buffer and 0.25% bupivicaine at a pH of 6.5.

### Infection of rhesus macaques and overview of study

The study timeline is shown in Figure 1. Three RhCMV seropositive Mamu A*01 positive rhesus macaques were intravenously infected with SIVmac251 (1000 TCID_50_) at time zero. Four months after SIV infection, ART was introduced (subcutaneous PMPA 20 mg/kg/day (Gilead Sciences, Inc) and oral D4T 1 mg/kg/day (Bristol-Meyers Squibb)) and continued until the end of the experiment. The macaques were followed for 5.6 months on ART for recovery of peripheral CD4^+ ^T cell count and viral load suppression. Nine and a half months after infection and 5.6 months after the initiation of ART, each monkey received 2 immunizations with SIV gag encoding DNA [19,21-23] intramuscularly (i.m.) (2 mg/injection), one in the left arm (triceps muscle) and one in the left leg (quadriceps muscle) and 2 immunizations with RhCMV pp65 encoding plasmid [20] (2 mg/injection) in the right arm and leg. After immunization, draining LN were sampled at two-time points for each animal by first removing inguinal LNs on each side followed by axillary LN removal from both sides (Macaque FH40 - D3 and D9, Macaque DD05 - D5 and D11, and Macaque CT64 - D7 and D14). On day 60 post immunization, LNs from both sides of each animal, draining SIV gag and RhCMV pp65 immunizations, were collected. Blood was collected every 2-3 weeks throughout the experiment. PBMC were isolated using Ficoll-diatrizoate gradient centrifugation and analyzed by flow cytometry or cryopreserved in 10% DMSO.

### Measurement of peripheral viral load and CD4+ T cell counts

Viral RNA in plasma was quantified by a bDNA signal amplification assay (Bayer Inc., version 4.0), specific for SIV, which has a threshold detection limit of 125 copies per ml of plasma [47].

CD4^+ ^T cell counts using whole blood collected in EDTA were analyzed with anti-CD3 (Clone SP34), anti-CD4 (Clone L200) and anti-CD8 (Clone SK1) fluorochrome-labeled monoclonal antibodies (Beckton-Dickinson) and white blood cell counts, as previously described [48].

### LN biopsy

LN biopsy collection and processing were performed as previously described [49]. Briefly, LNs were diced into small pieces using scalpel blades and then pressed through nylon mesh screens and triturated to generate single-cell suspensions. The single cell suspensions were divided into two parts; one was cryopreserved in 10% DMSO and stored in liquid nitrogen and one was frozen as a cell pellet.

### In vitro stimulation

Cryopreserved LNMCs or PBMCs were thawed and re-suspended in complete RPMI 1640 supplemented with 10% heat-inactivated serum (HyClone) and L-glutamine (Invitrogen) and rested for two hours at 37°C. Unless otherwise noted, cells were prepared at 1 × 10^6 ^cells/ml for *in vitro *stimulation. For characterizing the SIV- and RhCMV-specific T cell responses, LNMCs or PBMCs were incubated with either SIVmac239 gag 15-mer peptide pool with 11-amino acid overlap, 2 μg/ml each peptide (NIH AIDS Research and Reference Reagent Program) or rhesus CMV pp65 complete peptide pool (15-mers overlapping by 11 amino acids) [20] at 2 μg/ml for each peptide at 37°C for 6 hours in the presence of Golgi-Stop (0.7 μg/ml), Golgi-Plug (1 μg/ml), and 1 μg/ml of co-stimulatory antibodies anti-CD28 and anti-CD49d (BD Bioscience). Negative control with no stimulation and positive control with PMA (50 ng/ml) and Ionomycin (500 ng/ml) (Sigma-Aldrich) were used.

### Cell staining and analysis

After 6 hours of stimulation, cells were washed with washing buffer (PBS with 1% FBS, 0.09% NaN_3_) and stained with aqua blue dye (Invitrogen) and pre-titrated amounts of fluorochrome-conjugated surface staining antibodies (anti-CD4-PerCP Cy5.5, anti-CD8-FITC, anti-CD14-Pac Blue, anti-CD16-Pac Blue, anti-CD95-PE-Cy5, anti-CD20-Pac Blue (eBioscience), and anti-CD28-ECD (Beckman Coulter) and incubated at 4°C for 20 minutes. Cells were then washed and fixed in 250 μl BD Fixation/Permeabilization solution (BD Biosciences) for 20 minutes at 4°C. After fixation, cells were permeabilized with 1 × BD Perm/Wash buffer and stained with pre-titrated fluorochrome-conjugated antibodies (anti-CD3-APC-Cy7, anti-IL-2-PE, anti-IFN-γ-APC, and anti-TNF-α-PE-Cy7 (BD Biosciences) at 4°C for 45 minutes. Cells were then washed with Perm/Wash buffer and re-suspended in 300 μl PBS, 1% FBS.

Cells were analyzed on an LSR-II flow cytometer (BD Biosciences) equipped for the detection of 18 fluorescence parameters and 200,000 to 500,000 events were obtained. Flow Jo version 8.8.7. (Tree Star) was used to analyze the polychromatic flow data with the analytic gating performed as described [50,51].

### Tetramer analyses

PBMCs were stained with aqua blue; CD14-, CD16-, and CD20-pacific blue; CD3-Cy7-APC; CD8-FITC; and p11C (CTPYDINQM) tetramer-APC (Beckman Coulter) and analyzed on an LSR II flow cytometer.

### Quantitative PCR

Total RNA from LNMCs was isolated using Trizol according to manufacturer's instruction (Invitrogen) and subject to real-time (RT)-PCR on an ABI 7500 (Applied Biosystem). Doubly spliced (Tat), singly spliced (Vif), and unspliced (Gag) SIV RNA and the housekeeping gene GAPDH were analyzed. The primers and MGB probes for these genes were obtained from Applied Biosystems (Table 2). Changes in the expression of individual viral RNAs with GAPDH normalization were calculated utilizing delta cycle threshold (Δ*C_T_*) values.

**Table 2:** Primers and probes for real-time PCR

SIV doubly spliced	Forward: 5'- AGGCTAATACATCTTCTGCATCAAAC - 3'
	Reverse: 5'- GGGTCCTGTTGGGTATGAGTCTA - 3'
	Probe: 5' - CCACCCTCTTATTTCC - 3'
SIV singly spliced	Forward: 5'- AGAGGCCTCCGGTTGCA-3'
	Reverse: 5'- CCTTCCCCTTTCCACAATAGC-3'
	Probe: 5'-ACTGTGGAAGGGACC-3'
SIV unspliced	Forward: 5'- TTGCAGCACCCACAACCA-3'
	Reverse: 5'-TGATCCTGACGGCTCCCTAA-3'
	Probe: 5'- CTCCACAACAAGGACA-3'
IFN-γ	Forward: 5'- GTGTGGAGACCATCAAGGAAGAC-3'
	Reverse: 5'- CGACAGTTCAGCCATCACTTGGAT-3'
	Probe: 5'-ACTGACTCGAATGTCCAACGCAAAGC-3'
GAPDH	Forward: 5'-GGCATCCTGGGCTACACTGA-3'
	Reverse: 5'-AGGAGTGGGTGTCGCTGTTG-3'
	Probe: 5'- AGGTGGTCTCCTCTGAC -3'

Levels of IFN-γ mRNA were quantitated by RT-PCR against a standard curve derived from serial dilutions of in vitro made transcripts using specific primers and probe (Table 2). Copies of IFN-γ mRNA were expressed as per 1 μg of total RNA.

### Statistics

Mean, standard error of the mean, and student's *t-*test were performed using Microsoft Excel software. For the comparison of viral RNA in LNs draining SIV and RhCMV antigen immunization sites on the same day from the same animal, random effects models were used to adjust for the inherent correction between observations from the same animal. Relative changes were log-transformed for analyses in order to meet normality assumptions. SAS 9.2 was used for the analyses.

## Competing interests

The authors declare that they have no competing interests.

## Authors' contributions

HH performed the immunologic and virologic analyses and drafted the manuscript. LG performed immunologic analyses. PPA and AAL performed all animal manipulations and experimentation. JEC and PAB participated in its design and coordination. DW conceived of the study, participated in its design and coordination, and helped to draft the manuscript. All authors read and approved the final manuscript.
